# Supplementary material for: HIVprotI: an integrated web based platform for prediction and design of HIV proteins inhibitors
Source: J Cheminform. 2018 Mar 9;10:12. doi: 10.1186/s13321-018-0266-y (PMC5845081; doi:10.1186/s13321-018-0266-y)
Supplement: Supplementary file 2 — Additional file 2. Source code of HIVProtI web server. [file 13321_2018_266_MOESM2_ESM.zip › HIVprotI_Source-code/hivprotI/weka-3-6-6/doc/weka/classifiers/trees/m5/SplitEvaluate.html]

SplitEvaluate


---


|  |  |  |
| --- | --- | --- |
| **Overview** \| **Package** \| **Class** \| **Tree** \| **Deprecated** \| **Index** \| **Help** \| **Weka's home** | |  |
| **PREV CLASS**   **NEXT CLASS** | **FRAMES**    **NO FRAMES**     **All Classes** |  |
| SUMMARY: NESTED \| FIELD \| CONSTR \| METHOD | DETAIL: FIELD \| CONSTR \| METHOD |  |


---


## weka.classifiers.trees.m5 Interface SplitEvaluate

**All Known Implementing Classes:**: CorrelationSplitInfo, YongSplitInfo

---

``` public interface SplitEvaluate ```

Interface for objects that determine a split point on an attribute

**Version:**
:   $Revision: 1.3 $

**Author:**
:   Mark Hall (mhall@cs.waikato.ac.nz)

---

| **Method Summary** | |
| --- | --- |
| `void` | `attrSplit(int attr, Instances inst)`             Finds the best splitting point for an attribute in the instances |
| `SplitEvaluate` | `copy()`             makes a copy of the SplitEvaluate object |
| `double` | `maxImpurity()`             Returns the impurity of this split |
| `int` | `position()`             Returns the position of the split in the sorted values. |
| `int` | `splitAttr()`             Returns the attribute used in this split |
| `double` | `splitValue()`             Returns the split value |

**Method Detail**

### copy

```
SplitEvaluate copy()
                   throws java.lang.Exception
```

:   makes a copy of the SplitEvaluate object

    :   **Returns:**: a copy of the object **Throws:**: `java.lang.Exception`

---


### attrSplit

```
void attrSplit(int attr,
               Instances inst)
               throws java.lang.Exception
```

:   Finds the best splitting point for an attribute in the instances

    :   **Parameters:**: `attr` - the splitting attribute: `inst` - the instances **Throws:**: `java.lang.Exception` - if something goes wrong

---


### maxImpurity

```
double maxImpurity()
```

:   Returns the impurity of this split

    :   **Returns:**: the impurity of this split

---


### position

```
int position()
```

:   Returns the position of the split in the sorted values. -1 indicates that
    a split could not be found.

    :   **Returns:**: an `int` value

---


### splitAttr

```
int splitAttr()
```

:   Returns the attribute used in this split

    :   **Returns:**: the attribute used in this split

---


### splitValue

```
double splitValue()
```

:   Returns the split value

    :   **Returns:**: the split value


---


|  |  |  |
| --- | --- | --- |
| **Overview** \| **Package** \| **Class** \| **Tree** \| **Deprecated** \| **Index** \| **Help** \| **Weka's home** | |  |
| **PREV CLASS**   **NEXT CLASS** | **FRAMES**    **NO FRAMES**     **All Classes** |  |
| SUMMARY: NESTED \| FIELD \| CONSTR \| METHOD | DETAIL: FIELD \| CONSTR \| METHOD |  |


---
